# Morphological Asymmetry of the Superior Cervical Facets from C3 through C7 due to Degeneration

**DOI:** 10.1155/2017/5216087

**Published:** 2017-11-22

**Authors:** Nicolas Van Vlasselaer, Peter Van Roy, Erik Cattrysse

**Affiliations:** Experimental Anatomy Research Department (EXAN), Vrije Universiteit Brussel, Building B, Room B037, Laarbeeklaan 103, 1090 Brussel, Belgium

## Abstract

**Introduction:**

Knowledge about facet morphology has already been discussed extensively in literature but is limited regarding asymmetry and its relation to facet degeneration.

**Method:**

Facet dimensions, surface area, curvature, and degeneration of the superior facets were measured in 85 dried human vertebrae from the anatomical collection of the Vrije Universiteit Brussel. The vertebrae were analysed using the Microscribe G2X digitizer (Immersion Co., San Jose, CA) and a grading system for the evaluation of cervical facet degeneration. Coordinates were processed mathematically to evaluate articular tropism. The statistical analysis includes the paired *t*-test and the Pearson correlation.

**Results:**

On average, no systematic differences between the left and right facets were found concerning morphology and degeneration. However, there were significant differences regardless of the side-occurrence. There was a significant correlation between the dimensions of the total facet surface and the degree of degeneration but not for the recognizable joint surface.

**Conclusions:**

Facet tropism of the upper joint facets occurred often in the cervical spine but without side preference. A bigger difference in degeneration asymmetry was associated with a bigger difference in facet joint dimension asymmetry.

## 1. Introduction

The assumption of symmetry as often assumed in medical practices can be questioned. During examination and treatment of the cervical spine, it is assumed that the facet surfaces of the vertebrae are symmetrical. In order to achieve an absolutely symmetrical movement, neuromuscular control must also be symmetrical as well as the anatomical morphology.

Since assumptions and evidence of asymmetry of the cervical facet joints are commonly reported, full symmetrical motion within these segments cannot be taken for granted [1, 2].

Putti first defined left-right asymmetry of zygapophysial joints as articular tropism in 1927 [3]. A zygapophysial joint (or facet joint) can be morphologically described by its shape, size, orientation, curvature, implantation, and/or the presence of osteophytes, and, consequently, also their asymmetry [4].

Tropism may be of congenital origin or may be caused by degenerative deformations such as osteophytes or degenerative enlargement and can be accelerated as a result of rheumatoid diseases [5, 6]. These deformations could also come from adaptations to the passive stabilization system, when the active stabilization system works inadequate due to aging [7]. It is unclear whether asymmetry of the facet joints has an influence on the quality and quantity of the cervical movements.

Degenerative enlargement of the inferior facet surface of the atlas (C1) appears to be the highest cause for cervical articular tropism [4].

At the mid- and lower cervical spine, asymmetry of the surface area of the facet joints occurs frequently and asymmetry regarding orientation of these facets has been demonstrated as well [8–10].

Lee and Riew demonstrated that degeneration of the facet joints mainly occurs in the mid part, rather than in the lower part of the cervical spine [11].

The purpose of this research is to further investigate and quantify asymmetries of the cervical joints from C3 through C7 due to degeneration. Mainly the facet dimensions and curvature of the joints will be measured and the associated degree of degeneration and asymmetry will be analysed. This information should help to expand the knowledge of facet joints anatomy and improve insights into the possible consequences of this asymmetry due to degeneration on movements of the cervical spine.

## 2. Method

### 2.1. Data Collection

Cervical vertebrae from the anatomy collection at the Vrije Universiteit Brussel were used to make observations and measurements of the superior facets. The vertebrae came from donors between the ages of 50 and 90 years.

Vertebrae were not included in this study when there was damage to the facets due to the maceration process or when vertebral block formation, compression fracture, or other signs of trauma were observed.

A total of 85 cervical vertebrae were included in this study.

The morphology of the superior facets were collected by using a Microscribe G2X digitizer (Immersion Co., San Jose, CA).

**Figure 1:**
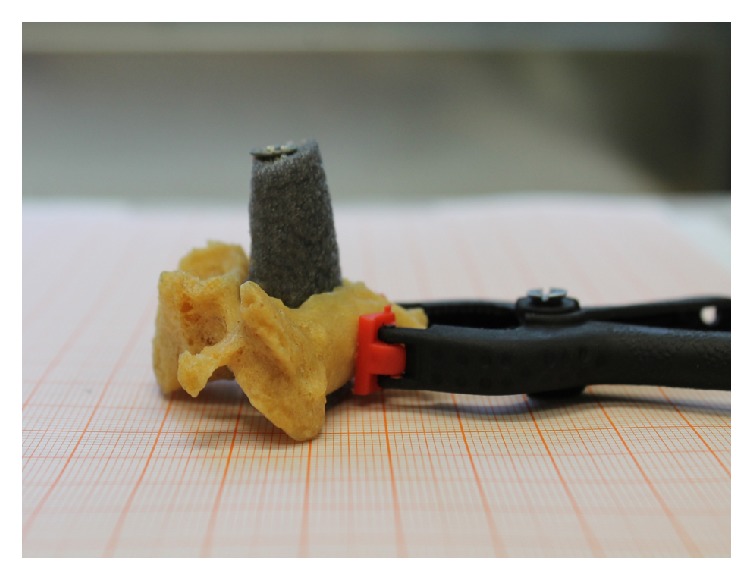
Fixation system on graph paper.

**Figure 2:**
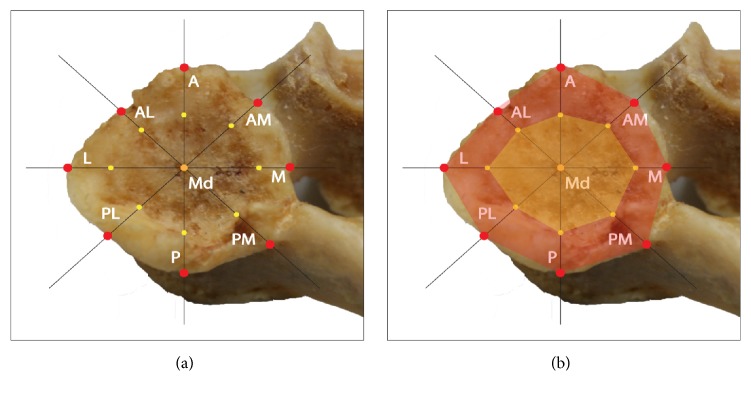
(a) Points registered with the Microscribe on the superior facet. A: anterior, AM: anteromedial, M: medial, PM: posteromedial, P: posterior, PL: posterolateral, L: lateral, AL: anterolateral, Md: center. (b) Red: total facet surfaces (TFS), Yellow: recognizable joint surface (RJS).

Vertebrae were fixed (Figure 1) and 37 coordinates were registered on each specimen. Three of these served to build a local bone embedded reference frame. Additionally, 17 points on each facet surface (Figure 2(a)) were digitized.

Using a mathematical routine, the dimensions of the total facet surface (TFS, red area in Figure 2(b)) and the recognizable joint surface (RJS, yellow area in Figure 2(b)) of each superior facet could be calculated. The curvature could be calculated by measuring perpendicular distance from the center (Md) to the line connecting the anterior (A) and posterior (P) point. This was repeated for the line connecting the lateral (L) and medial (M) point [2]. During the maceration process the removed cartilage leaves a circular mark on the cortical bone of the facet. This circular mark was defined as the RJS and could be compared to the epiphyseal ring described on the vertebral endplate [12].

The existing scales for determining degeneration of the facets only measured parts of the degeneration process. Therefore, a more comprehensive scale was developed to provide a measurement tool that takes all aspects of degeneration in consideration. This gradation scale for assessing cervical facet degeneration on dry bones was developed, inspired on three existing grading systems by Kellgren et al. (1963), Wedel et al. (1978), and Walraevens et al. (2009) [13–15], each of them describing specific aspects of the degenerative process. By making the sum of the points from three subscales (“surface,” “edge-osteophytes,” and “edge-hypertrophy”), the gradation system gives a score from 0 to 9 (Table 1). Based on the total score from Table 1 a final grade from 0 to 4 could be assigned, thus, depicting the general degree of facet degeneration (Table 2).

### 2.2. Statistical Analysis

The new gradation system was tested for intra- and interobserver reliability using the Intraclass Correlation Coefficient (ICC).

Data concerning facet dimensions, surface, and curvature were checked for normality with QQ-plots. For all of the data, the mean and standard deviation (SD) were calculated. Additionally the mean and the SD of the absolute differences between the left and the right superior facet were calculated. Secondly, the left-right data were analysed by a paired samples *t*-test. The data from left and right sides were tested two-sided in order to identify level based facet asymmetry. To examine the asymmetry per vertebra, irrespective of occurrence on the left or the right side, the data was redistributed into two groups, namely, highest value per vertebra and lowest per vertebra, that is, regardless of the side-occurrence.

Secondly, Pearson correlation coefficients were calculated to assess the relationship between asymmetry in facet degeneration and asymmetry in facet morphology.

### 2.3. Validity and Reliability

Validity and reliability of the Microscribe were assessed in previous studies.

Masharawi et al. (2004) tested its validity and reliability in the thoracic and lumbar spine [16]. Their intraobserver and interobserver reliability both scored high (>99.5% and >97.5%) for all coordinates, regardless of the anatomical landmark and the time interval between measurements.

The validity of the device was high (98%–100%) when compared to a 0.01 mm accurate digital callipers (Mitutoyo Co.), a standard three-dimensional scanner, and a digital goniometer (ProSmart Co.).

The intraobserver reliability for the use of the Microscribe in this study was confirmed by digitizing a set of ten vertebrae by the same researcher twice. The interobserver reliability was tested by digitizing the same vertebrae by a second researcher. From this data, the dimensions were calculated and an ICC was performed.

The new gradation system was tested on intraobserver reliability by retesting 40 vertebrae, with a time interval of four weeks. The interobserver reliability was tested by reevaluating 40 vertebrae by a second researcher.

## 3. Results

### 3.1. Validity and Reliability

Intra- and interobserver reliability of the overall grading system for the evaluation of facet degeneration scored well with ICCs equaling 0.91 for intraobserver reliability and 0.89 for interobserver reliability.

Here subscale 1 (surface) scored an ICC of 0.802; subscale 2 (edge-osteophytes) 0.817; and subscale 3 (edge-hypertrophy) 0.646, measured on two sets of 40 vertebrae.

The intra- and interobserver reliability for the use of the Microscribe in this study scored well with ICCs, respectively: 0.87 and 0.78, based on the 95% confident interval. These results are a bit lower than those from the study of Masharawi et al. (2004) [16] but still indicate a good reliability.

### 3.2. Asymmetry in Facet Dimensions and Curvature

The mean values and SD for the calculations of the superior facet are demonstrated in the first four columns of Table 3, while the mean differences between the smaller and larger superior facet of the vertebra are shown in the last two columns. All variables showed normally distributed data.

#### 3.2.1. Dimensions and Surface Area

The mean facet dimensions for both left and right superior facets in this study were calculated as absolute distances between 3D coordinates digitized by the Microscribe. The dimensions, that is. based on the four calculated diameters (Table 3) of the TFS were in the same order of magnitude (no significant differences). This indicates a circular shape of the facets. For the RJS, the mediolateral distances on both left and right sides were statistically bigger (<0,001) than the anteroposterior distances. This indicates a minimally more ellipsoid shape of the RJS. The means and SDs are listed in Table 3.

Using a paired sample *t*-test, the left and right superior facets of each vertebra were compared. Only for the measurements “facet depth in anterior to posterior direction” at the TFS (m = 0.31 mm, SD = 0.72 mm) and “anterolateral-posteromedial distance” at the RJS (m = 1.51 mm, SD = 2.28 mm) a significant difference between the left and right superior facet was demonstrated. For all other measurements, the average difference was around 0 mm. However large standard deviations occurred.

Comparing the surface area of the superior facets showed a higher value for the left superior facet in 54% of the vertebrae. Again this difference between the left and right side was not significant (*p* = 0.567).

The differences between the left and right sides were calculated by subtracting the absolute values of the left and right dimensions. The means and SDs are listed in Table 3.

Taking the largest determined area of each vertebra, regardless of whether it occurred on the left or the right side, as well as the smallest, thus regardless of side-occurrence, a new data set was created. Using a paired sample *t*-test significant differences were shown for the measurements on both the TFS and on the RJS. These significant differences are also shown in Table 3.

#### 3.2.2. Curvature

Based on the perpendicular distance from the facet joint center to the line connecting the anterior and posterior point on the facet border, the depth of the facet could be estimated. The ratio was calculated between the depth/height and the diameter to express the curvature of the facet [2]. In this way both the facet depth and the size of the facet itself were taken into account to analyse the joint surface curvature. No convex curvature of any superior facet could be demonstrated. Most facets either were flat or demonstrated a small concavity ratio and an average facet depth of 0.83 mm (SD = 0.52 mm) for the TFA and 0.67 mm (SD = 0.34 mm) for the RJS in the AP direction and 0.91 mm (SD = 0.54) and 0.75 mm (SD = 0.39 mm) in the ML direction.

**Figure 3:**
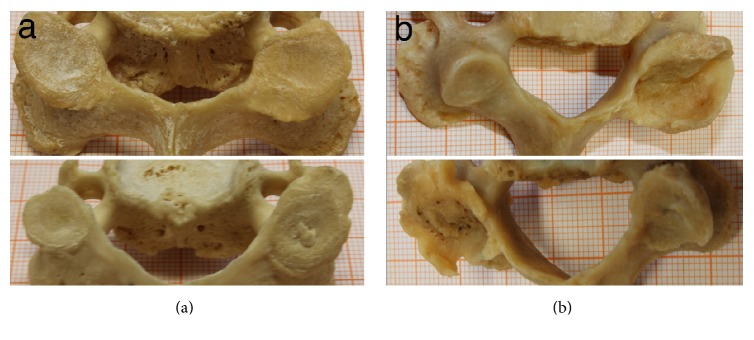
(a) Examples of facets showing a flattened pancake shape degeneration. (b) Examples of facets showing a cup shape degeneration.

Figure 3 shows an example of vertebrae having facet degeneration that forms a flattened pancake shape (Figure 3(a)) and facets that are degenerated into a cup shape (Figure 3(b)) [4].

### 3.3. Asymmetry in Facet Degeneration

The average score for the degree of degeneration of the superior facets was 2.87 (SD = 2.15), which corresponds to mild degeneration. When comparing the left and right side, no significant differences were found. With 43,5% being more degenerative on the right side and 35,3% on the left, 21,2% showed the same degree of degeneration (*p* = 0.952).

Regardless of the side-occurrence, a significant difference in degeneration is demonstrated between the two superior facets of the same vertebra, regardless of whether it occurred on the left or on the right side (*p* < 0.001). Comparing degeneration regardless of side-occurrence showed that the mean degree of degeneration of the most degenerated facet on each vertebra was m = 4.0 and SD = 2.20, while the mean degree of degeneration of the least degenerated facet was m = 1.74, SD = 1.37.

### 3.4. Relationship between Facet Dimensions and Degeneration

The Pearson correlation coefficient was calculated to assess the relationship between the asymmetry in facet degeneration and the asymmetry in facet morphology of the superior facets joints (Table 4). A significant positive correlation was wound for all the variables concerning the size of the TFS and the degree of degeneration.

For the measurements performed on the RJS, only significant positive correlations were found for the anteromedial-posterolateral distance (AMPL) and degeneration (*r* = 0.319, *p* = 0.003), as well as the anterolateral-posteromedial distance (ALPM) and degeneration (*r* = 0.391, *p* < 0.001).

**Figure 4:**
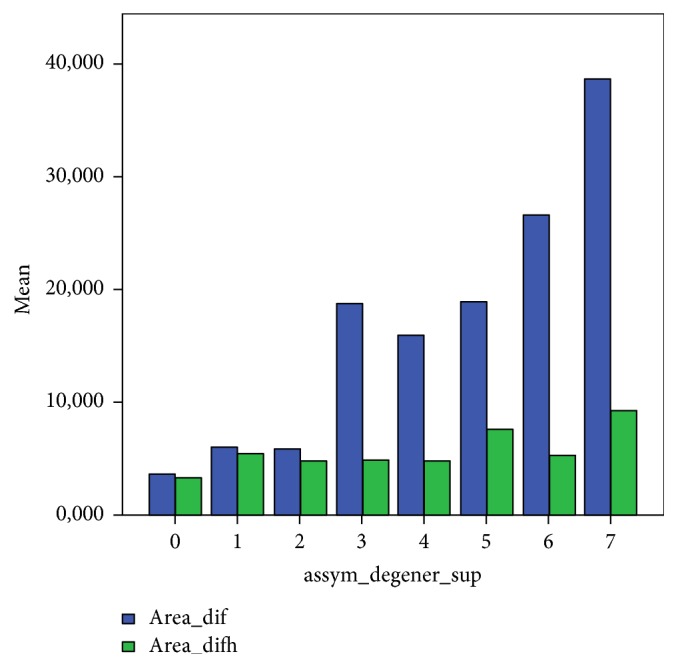
*x*-axis: asymmetry in degeneration. *y*-axis: asymmetry in surface area. Blue: total facet surface (TFS); green: recognizable joint surface (RJS).

Figure 4 shows the correlations between the degree of degeneration and the surface area of the TFS and the RJS.

Generally, there was a strong positive correlation between the difference in degeneration and the difference in dimensions and surface measurements on the TFS, *r*: 0,760 (*p* < 0.01), but not in the measurements on the RJS, *r*: 0,191 (*p* = 0.08).

## 4. Discussion

The aim of this study was to examine the morphology and asymmetry of the superior cervical facets regarding the dimensions, curvature, and degeneration. By using a more comprehensive scale for the assessment of degeneration, more aspects of the degenerative process could be taken into consideration. Also, the correlation between the facet morphology and the degree of degeneration was addressed.

### 4.1. Grading System

During the visual assessment of facet degeneration on dried vertebrae, the assessment of osteophytes was not obvious because the contours of the facet joints may be physiologically irregular. These irregularities do not implicitly need to be considered as a sign of osteophytic formation [9]. This was taken into account when creating the gradation system. The confusion between osteophytes (subscale 2) and hypertrophy (subscale 3) also seemed to be an obstacle. Therefore interobserver reliability was taken into account for each of the three subscales. The lower score of subscale 3 seems to be corrected in the total score because of the confusion with subscale 2, which makes this grading system a good tool for measuring the total degree of degeneration on dried cervical vertebrae.

### 4.2. Morphology

Mean values and SDs from this study correspond well to the values presented by Panjabi et al., 1993 (10.2 to 13.9 mm) (facet widths), and Yoganandan et al., 2003. Compared with the data reported by Milne (1991) (8.5 mm to 10.4 mm), this study shows slightly higher facet width values. Also, Francis (1955) described slightly lower values from 10.1 mm to 12.3 mm in men and 9.9 mm to 11.7 mm in women [17–19].

The gap is defined by Yoganandan et al., 2003, as the distance from the most ventral or distal part of the facet joint to the site where the cartilage begins; thus the lack of cartilage covering [18] could in this study also be demonstrated by the difference in dimensions between the points measured on the TFS and the points on the RJS (see Figure 2(b)). On average, this difference was 3.19 mm (SD = 1.32 mm) for the anteroposterior distance. These results correspond to the results of Yoganandan et al. where the average was 3,71 mm (SD = 0,74) which confirms that the RJS matches the area normally covered by cartilage in vivo.

The ratio depth versus anteroposterior diameter was very small (0.08 and 0.05), which made it clear that the curvature is slightly concave but tends to be very flat. Also, Pal and Routal (1999) emphasized that the facet surfaces at C3–C6 are mainly flat [20]. Womack et al. (2008) showed a slight convexity when examining facet surfaces at C3 to C7 vertebrae from seven cadavers, but in their study the cartilage was still present [21].

The fact that the curvature on the TFS was greater than that on the RJS could be due to osteophytic formation and hypertrophy that grows in the direction of the joint capsule, creating a kind of cup shape. We found this type of degeneration in 17 out of 85 vertebrae. Due to the congruence between joint surfaces, this form of degeneration might have serious effects on the mobility of the vertebra.

On the other hand, some vertebrae exhibited facet hypertrophy, giving the facet a more flattened (pancake shape like) curvature [4], which could increase mobility (in 19 of the 85 vertebrae) (Figure 3). One can therefore wonder if degeneration has a positive effect on stability at the expense of a reduction in mobility and vice versa. These hypotheses are yet to be studied.

### 4.3. Asymmetry in Facet Dimensions, Degeneration, and the Relation between Them

There were no significant differences between left and right facets. However, there is a significant difference between the facets individually. On one side, a larger area may be present than on the other side for both the TFS and the RJS regardless to left or right side.

The question can be asked whether this difference in area results in kinematic differences and whether an enlargement of a facet on one side also increases translation over this surface relative to the other side, thus resulting in motion asymmetry.

Just like the asymmetry in dimensions, there was no preference for the left or right side regarding facet degeneration.

However, regardless of the side-occurrence there is a large asymmetry of degeneration. Thus, it can be hypothesized that asymmetry in degeneration could cause unilateral complaints.

A clear correlation could be demonstrated between the difference in degeneration and the difference in facet dimensions at the TFS, but not for the RJS.

From this it could be deduced that when a facet is affected by degeneration, the TFS will expand and become larger in all directions (mainly in the AP direction) but the RJS will be spared and does not seem to increase or in some cases even reduces.

The positive correlation shown for the degree of degeneration and curvature of the facet for the TJS can be explained by the previously mentioned cups shape as a result of degeneration.

As a limitation of this study, we should mention that the vertebrae were collected over the years and the age, gender, and vertebral level were not registered. In future research a similar study should be conducted labeling the vertebrae with age, gender, and vertebral level data. Preforming additional studies on 3D imaging will allow studying larger data sets in relation to clinical background.

## 5. Conclusion

The morphologic measurements correspond well to data from previous research.

As far as asymmetry in morphology is concerned, no preference can be given to the left or right facet. However, there are major differences regardless of the side-occurrence. Also for degeneration, there is on average no preference for the left or right facet, but there are major differences regardless of the side-occurrence. For example, one side can show very strong degeneration, while the other side is not affected.

When a facet exhibits a higher degree of degeneration, this often results in a larger total facet surface, but the recognizable joint surface remains fairly constant or even reduced in some cases.

Regarding curvature, a slight increase in curvature of the TFS was present with higher degrees of degeneration (*r* = 0.284, *n* = 85, *p* = 0.009), whereas the RJS does not exhibit the same correlation or is even a little flatter, although not significantly (*r* = −0.03, *n* = 85, *p* = 0.81).

Currently, we can only hypothetically state that asymmetry in the degree of degeneration and morphology of the superior facets could affect unilateral complaints, movement restrictions, and stability problems.

Further research should examine the asymmetry shown in this study in vivo, possibly relying on medical imaging and kinematical analysis related to clinical presentation.

## Figures and Tables

**Table 1:** Grading system for evaluation of cervical facet degeneration on dried vertebrae, consisting of three subscales.

(1) Surface	
Smooth	0 points
Irregularities	1 point
Irregularities with small perforations	2 points
Serious irregularities with large perforations (>3 mm^2^)	3 points
(2) Edge – osteophytes	
No osteophytes, smooth edge	0 points
Small osteophytes, rougher edge	1 point
Medium to large osteophyte	2 points
Several large osteophytes	3 points
(3) Edge – hypertrophy	
No hypertrophy	0 points
Hypertrophy on one edge	1 point
Multi-edge hypertrophy	2 points
Full pancake formation	3 points

**Table 2:** General degree of facet degeneration (sum of the three subscales).

Grade 0	0-1 points	No degeneration
Grade 1	2-3 points	Mild degeneration
Grade 2	4-5 points	Moderate degeneration
Grade 3	6-7 points	Advanced degeneration
Grade 4	8-9 points	Severe degeneration

**Table 3:** The mean values and SDs for the calculations of the superior facets from C3 to C7 on a total of 85 vertebrae and the mean differences between the smaller and larger superior facet from C3 to C7 on a total of 85 vertebrae.

Morphological characteristics and differences Mean (SD) *N* = 85	Total facet surface Left	Total facet surface Right	Recognizable joint surface Left	Recognizable joint surface Right	Total facet surface Smaller vs Larger	Recognizable joint surface Smaller vs Larger
Dimensions and surface area (mm)						
Anteroposterior distance	12,34 (2,66)	12,38 (3,18)	9,1 (1,52)	9,24 (1,68)	2,47 (2,26)^*∗∗*^	1,11 (0,86)^*∗∗*^
Mediolateral distance	12,55 (1,69)	12,26 (1,99)	10,28 (1,42)	10,18 (1,44)	1,61 (1,43)^*∗∗*^	0,98 (0,75)^*∗∗*^
Anteromedial-posterolateral distance	11,81 (1,84)	11,62 (2,50)	9,51 (1,38)	9,33 (1,67)	1,9 (1,61)^*∗∗*^	0,99 (0,91)^*∗∗*^
Anterolateral-posteromedial distance	12,84 (2,23)	13,12 (2,96)	12,01 (1,61)	10,51 (1,83)	2,18 (2,18)^*∗∗*^	2,17 (1,64)^*∗∗*^
Surface area (mm^2^)	78,21 (12,02)	77,42 (14,99)	60,87 (7,46)	60,98 (8,68)	11,51 (10,9)^*∗∗*^	5,05 (4,03)^*∗∗*^

Curvature (mm/ratio)						
Facet depth in anteroposterior direction	0,99 (0,55)	0,67 (0,48)	0,7 (0,39)	0,63 (0,31)	0,6 (0,5)^*∗∗*^	0,32 (0,3)^*∗∗*^
Facet depth in anteroposterior direction/Anteroposterior distance	0,08 (0,04)	0,05 (0,03)	0,08 (0,05)	0,07 (0,03)	0,05 (0,04)^*∗∗*^	0,04 (0,03)^*∗∗*^
Facet depth in mediolateral direction	0,86 (0,47)	0,95 (0,60)	0,74 (0,41)	0,75 (0,37)	0,47 (0,44)^*∗∗*^	0,3 (0,25)^*∗∗*^
Facet depth in mediolateral direction/Mediolateral distance	0,07 (0,04)	0,08 (0,04)	0,08 (0,04)	0,08 (0,04)	0,04 (0,03)^*∗∗*^	0,03 (0,03)^*∗∗*^

Significance level: ^*∗∗*^*α* < 0,001.

**Table 4:** Relationship between the asymmetry in facet degeneration and the asymmetry in facet morphology of the superior facets joints C3–C7.

Correlations between difference in morphology L-R and difference in degeneration L-R	Total facet surface Pearson Correlation (*r*) Significance (2-tailed) (*p*) *N* = 85	Recognizable joint surface Pearson Correlation (*r*) Significance (2-tailed) (*p*) *N* = 85
Difference in dimensions and difference in degeneration		
Anteroposterior distance	0,712^*∗∗*^ (*p* < 0.01)	0,207 (*p* = 0.058)
Mediolateral distance	0,584^*∗∗*^ (*p* < 0.01)	0,195 (*p* = 0.074)
Anteromedial-posterolateral distance	0,692^*∗∗*^ (*p* < 0.01)	0,319^*∗∗*^ (*p* = 0.003)
Anterolateral-posteromedial distance	0,634^*∗∗*^ (*p* < 0.01)	0,391^*∗∗*^ (*p* < 0.01)
Surface area	0,760^*∗∗*^ (*p* < 0.01)	0,191 (*p* = 0.08)

Difference in curvature and difference in degeneration		
Facet depth in anteroposterior direction	0,029 (*p* = 0.793)	−0,045 (*p* = 0.685)
Facet depth in anteroposterior direction/anteroposterior distance	−0,054 (*p* = 0.623)	−0,042 (*p* = 0.703)
Facet depth in mediolateral direction	0,226^*∗*^ (*p* = 0.038)	−0,026 (*p* = 0.815)
Facet depth in mediolateral direction/mediolateral distance	0,284^*∗∗*^ (*p* < 0.01)	0,013 (*p* = 0.908)

Significance level: ^*∗*^*α* < 0.05/^*∗∗*^*α* < 0,001.
